# Splenic accumulation of intact *Plasmodium ovale sensu lato*-infected red blood cells in a patient presenting with splenic rupture

**DOI:** 10.1371/journal.pntd.0013897

**Published:** 2026-01-08

**Authors:** Ghania Benabdelmoumen, Oula Itani, Valentin Joste, Sandrine Houzé, David Hardy, Florent Bavozet, Moussa Sylla, Steven Kho, Nicholas M. Anstey, Paul Henri Consigny, Abdoulaye Sissoko, Pierre Buffet

**Affiliations:** 1 Institut Pasteur, Centre Médical, Centre d’Infectiologie Necker Pasteur, Paris, France; 2 Université Paris Cité, Inserm, EFS, BIGR U1134, Paris, France; 3 Institut Pasteur, Rate-Infection-Parasites Unit, Paris, France; 4 Université Paris Cité, IRD, MERIT, Paris, France; 5 Centre National de Référence du Paludisme, AP-HP, Hôpital Bichat - Claude-Bernard, Paris, France; 6 Institut Pasteur, Experimental Neuropathology Unit, Paris, France; 7 Médecine Intensive Réanimation, CH de Dreux, Dreux, France; 8 Chirurgie viscérale, digestive et métabolique, CH de Dreux, Dreux, France; 9 Global and Tropical Health Division, Menzies School of Health Research and Charles Darwin University, Darwin, Northern Territory, Australia; University of the Witwatersrand Johannesburg, SOUTH AFRICA

## Abstract

A 35-year-old man had splenic rupture just after starting antimalarial treatment with atovaquone-proguanil followed by artesunate for acute *Plasmodium ovale sensu lato* infection. Spleen histology showed a 10-fold accumulation of intact *P. ovale s.l.*-infected erythrocytes in the spleen parenchyma compared to the general circulation. Infected erythrocytes also accumulated in small extra-splenic blood vessels, suggesting cytoadherence.

## Author summary

Spontaneous rupture of the spleen is an unusual but severe complication of malaria. Here, we present the case of an otherwise healthy 35-year-old man who presented with splenic rupture hours after starting antimalarial treatment for an acute *Plasmodium ovale sensu lato.* infection. The spleen is known to be a major site for parasite destruction in malaria, but recent observations have shown that intact *P. falciparum-* and *P. vivax*-infected red blood cells accumulate in the spleen. In our reported case, spleen histology showed a 10-fold accumulation of intact *P. ovale s.l.*-infected erythrocytes in the spleen parenchyma compared to the general circulation, suggesting that intact *P. ovale s.l.* similarly accumulates in the spleen tissue. We also found infected erythrocytes accumulated in small extra-splenic blood vessels, suggesting cytoadherence. Our case report reinforces the importance of carrying out further studies to better understand ovale malaria pathogenesis and potential clinical complications.

## Introduction

Spontaneous rupture of the spleen is an unusual but severe complication of malaria [1], the pathogenesis of which remains poorly understood [1]. Few cases of splenic rupture have been reported in *Plasmodium ovale sensu lato* [1–6]. Here, we describe non-traumatic spleen rupture during acute *P. ovale s.l.* malaria infection that led to splenectomy, and present the results of the histological analysis of the ruptured spleen.

## Case Report

A previously healthy 35-year-old French man, living in Dakar, Senegal, from 2021 to 2023, travelled to Abéché and Adré, Chad, in May 2023. Whereas he took no prophylaxis against malaria in Senegal, an atovaquone/proguanil regimen was correctly followed in Chad. In August 2023, while still in Chad, he presented with fever and headaches. His blood smear was reported as positive for *Plasmodium falciparum*, although the falciparum rapid diagnostic test (RDT) was negative. He was properly treated with artemether/lumefantrine and symptoms resolved. Follow-up ensued in Paris, France, at day 28: the patient was asymptomatic and both a blood smear and a RDT were negative for malaria parasites or antigens. He then returned to Dakar but travelled back to France in December. In January 2024, he developped fever up to 39°C, headaches and nausea. Malaria testing was done three days later when there was recurrent fever and vomiting, but microscopy was delayed until day 7 of illness. The blood smear was positive for *P. ovale s.l.* with 0.9% parasitemia and the patient presented to hospital. He was normal upon clinical examination but laboratory testing showed thrombocytopenia (56x10^9^/L). He was inappropriately discharged with primaquine for 14 days as the only prescribed antimalarial treatment, but he did not in fact start treatment. Because of recurrent fever, a different doctor was consulted on day 9 of illness, who prescribed atovaquone/proguanil 250/100mg x4 for 3 days. Four hours after taking his first dose on day 10, the patient became acutely unwell, with loss of consciousness, and he was transferred to hospital. On arrival, he was pale with signs of dehydration. His Glasgow Coma Score was 15 = responsive, blood pressure 59/32 mmHg, heart rate 97 beats/min and oxygen saturation 100% in room air. On palpation, he had subdiaphragmatic tenderness and abdominal guarding. Computed tomography scanning showed splenic rupture with moderate hemoperitoneum and a peri-splenic sentinel clot. The patient was admitted to intensive care, where the hemorrhagic shock was controlled by intravenous fluids, noradrenaline, and blood and plasma transfusion, while malaria was treated with artesunate. He underwent laparoscopic splenectomy, 14 hours after commencement of atovaquone/proguanil therapy and 2 hours of artesunate administration. The outcome was favorable, with noradrenalin discontinued on day 1 post-splenectomy. Artesunate was switched to dihydroartemisinin-piperaquine for 3 days on day 4. The patient was discharged on day 10 post-splenectomy with penicillin V 1 million IU bd for 2 years, aspirin 75 mg daily for 3 weeks and post-splenectomy vaccinations (pneumococcal, meningococcal and seasonal influenza). He was advised against visiting malaria endemic areas again.

**Fig 1:**
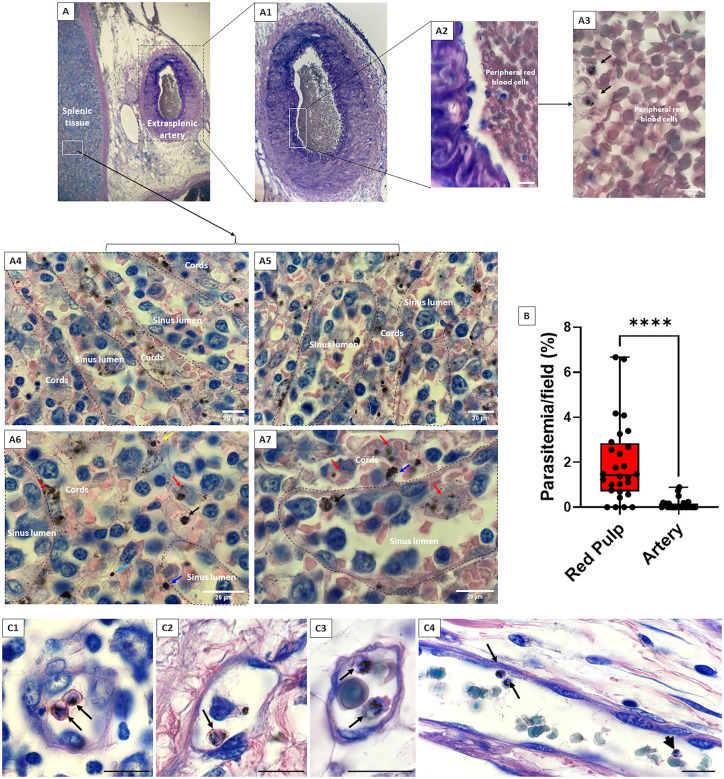
Splenic accumulation of *Plasmodium ovale sensu lato* (*s.l.*)-infected red blood cells. **(A)** Histological analysis on Giemsa-stained spleen fragments from the patient, displaying: **A1**–**A3.** an extra-splenic artery allowing the quantification of peripheral parasitemia; **A2:** scale bar = 20µm; **A4**–**A7.** splenic tissue, especially red pulp (sinus lumina and cords) showing intact *Plasmodium ovale s.l.*-infected red blood cell (slanted black arrow, **A6**–**A7**), macrophage containing parasite element (slanted red arrow), extraerythrocytic pyknotic form (slanted light blue arrow), intraerythrocytic pyknotic form (slanted yellow arrow), indeterminate parasite form (slanted blue arrow). Pyknotic forms were not included in parasite counts. **(B)** The proportion of **intact *P. ovale s.l.*-infected red blood cells** (slanted black arrow) per field was quantified in the red pulp (28 fields) and in the artery (27 fields), showing exclusively mature multinucleated forms, the Mann-Whitney test was performed (**** means *p* < 0.0001). **(C1**–**C4).** Images of extra-splenic small blood vessels suggesting the occasional cytoadherence of intact *P. ovale s.l.*-infected red blood cells (scale bar = 20µm).

On macroscopic examination, the 14x11x6 cm and 313 g spleen displayed no macroscopic clot. Microscopic examination of hematoxylin eosin saffron-stained sections at x400 magnification revealed red pulp congestion with erythrophagocytosis including macrophages displaying phagocytosed hemozoin pigment, and hyperplasia of the white pulp. Giemsa-stained sections screened at x1000 magnification enabled the quantification of infected and uninfected red blood cells (RBC) as has been described [7]. Non-phagocytosed, morphologically intact, parasitized RBC (excluding pyknotic forms) were observed in densities ten times higher in the spleen red pulp (Fig 1A4–1A7 and 1B) than in a large extra-splenic artery (Fig 1A1–1A3) (1.95 ± 0.02% vs 0.19 ± 0.05%, *p < 0.0001,* Fig 1B). The concentration of intact *P. ovale s.l.*-infected RBC was 66 times higher in small extra-splenic vessels (Fig 1C1–1C4) than in the large artery (12.62 ± 0.17% vs 0.19 ± 0.05%), and 6 times higher than in splenic red pulp (12.62 ± 0.17% vs 1.95 ± 0.02%), not shown.

PCR confirmed the microscopic diagnosis of malaria. Genomic DNA (gDNA) was extracted from paraffin-embedded spleen tissue using a QIAamp DNA mini kit (Qiagen, Germany). In-house qPCR were used to distinguish *P. o. curtisi* from *P. o. wallikeri* [8] (limit of detection ~5 p/µL) using 5 µL of gDNA and the Luna Universal Probe qPCR Master Mix (New England BioLabs Inc., Massachusetts). Given a recent history of *P. falciparum* malaria, we also searched for *P. falciparum* gDNA [9] (limit of detection ~0.1 p/µL). Briefly, 5 µL of gDNA were amplified with 400 nM of F and R primers, 250 nM of probe and 1x Luna Universal Probe qPCR Master Mix with the following program: 1 min at 95°C, 45 cycles of 15 sec at 95°C and 45 sec at 50.7°C. Results confirmed the presence of *P. o. wallikeri* in the spleen, with a Ct value of 36 (however, the name of this parasite might change when the current taxonomic confusion which prevails is resolved [10]). The *P. falciparum* qPCR, performed in triplicate, was negative.

## Discussion

The spleen is a major site for parasite destruction in malaria, but a more complex picture has emerged with recent observations from splenectomized subjects in malaria-endemic Indonesia, showing that intact *P. falciparum-* and *Plasmodium vivax*-infected RBC [7] accumulate in the spleen. Using the same validated quantification at high magnification of parasitized RBC on histological sections, we show here that non-phagocytosed RBC containing morphologically intact *P. ovale s.l.* parasite similarly accumulate in the spleen tissue compared to an extra-splenic artery present (by chance) in the tissue block. qPCR performed on DNA extracted from the spleen confirmed a *P. o. wallikeri* infection. This finding is not unexpected, as this species has been more frequently associated with severe clinical manifestations of malaria compared to *P. o. curtisi* [11]. RBC observed in this artery are a robust surrogate for others present in the general circulation at the time of splenectomy. By showing accumulation, our observation reported here for *P. ovale s.l.* is broadly consistent with the previous observations of splenic accumulation during asymptomatic *P. falciparum* or *P. vivax* infections [7,12]. The 10-fold accumulation observed here is less intense, however, than the 289- and 3590-fold accumulations of *P. falciparum-* and *P. vivax*-infected RBC in predominantly chronic infections in Indonesia. With *P. ovale s.l.* displaying similar biology to *P. vivax*, including the strict requirement for young reticulocytes that are enriched in the spleen [7], weaker splenic tropism in *P. ovale s.l.* infection is unlikely. The phase of Infection and indication for splenectomy in this case, namely, malaria-induced spleen rupture precipitated by *P. ovale s.l.*, versus post-trauma splenectomy with incidental chronic asymptomatic infection in Indonesia [7], may explain the difference in the level of splenic parasite occurrence observed in the two situations. In other words, the acute *P. ovale s.l.* malaria attack likely involved greater circulating parasitemia, as compared to the subacute/chronic *P. vivax* and *P. falciparum* infections in Indonesia, with lower, mostly subpatent peripheral parasitemia. Recent observations in *P. vivax*-infected subjects do indeed suggest that the ratio of parasite biomass accumulating in the spleen to that in circulation is lower in acute symptomatic than in chronic asymptomatic infections (Kho et al. 9^th^ International Conference on *Plasmodium vivax* Research (ICPvR), 2025).

Also, our estimation of the magnitude of splenic accumulation in *P. ovale s.l.* malaria may be conservative, with pyknotic forms not included in parasite counts. Atovaquone-proguanil and artesunate administered before splenectomy are unlikely to have markedly impacted the accumulation. Atovaquone-proguanil is indeed associated with slow parasite clearance [13] and, after starting with artemisinin derivatives, it takes at least 4–6 hours for the (then fast) parasite clearance to be triggered [14].

In summary, while several processes may have influenced its magnitude, parasite accumulation was marked enough in this case for *P. ovale s.l.* to be added to the list of *Plasmodium* species that have a spontaneous splenic tropism [15]. How and with what benefit these intact infected RBC accumulate in the spleen has not been fully elucidated yet, although the innate mechanical retention of ring parasite forms, observed in human spleens perfused *ex-vivo* [16], may play a triggering role for *P. falciparum* splenic retention. The selective affinity of *P. vivax* and *P. ovale s.l.* for immature reticulocytes, and the splenic accumulation of these RBC precursors [7], point to a selective advantage conferred to these species by splenic tropism. How the cryptic intrasplenic cycle of these splenotropic *Plasmodium* species contributes to their persistence, the emergence of parasite resistance to drugs, and may hamper malaria elimination efforts, is under intense scrutiny.

In addition to splenic tropism, we also observed intense accumulation (66-fold) of intact infected RBC in extra-splenic small vessels, suggesting adherence of *P. ovale s.l.-*infected RBC to endothelial cells. Such cytoadherence is central in the life cycle of *P. falciparum* and the pathogenesis of severe infections by this species. Cytoadherence, of unknown biological and pathogenic impact, has been also described in *P. vivax* malaria [17,18], but never to our knowledge for *P. ovale s.l*. infection. The parasite persistence observation is consistent with splenic rupture not being the only serious outcome induced by *P. ovale s.l.*; a systematic review points to other fatal complications [19]. The PCR results likely exclude a co-infection with *P. falciparum,* but the splenic tissue used for DNA extraction was paraffin-embedded, which reduces the quality and quantity of DNA obtained. We cannot therefore totally exclude the possibility of the presence of a low biomass of *P. falciparum* in the blood vessels.

The pathogenesis of non-traumatic spleen rupture in malaria is not fully understood. Intense congestion with predominantly uninfected RBC, which contributes to malarial anemia [20], is probably a major underlying process. We found 5 previous reports of splenic rupture following *P. ovale s.l.* infection [2–6] (S1 Table), 4 of them in non-immune travelers, and 1 in a migrant, all infected in sub-Saharan Africa. Although splenic rupture is sometimes the presenting feature of malaria, our patient was symptomatic at least for 10 days before effective treatment and the rupture, despite multiple medical encounters. In addition to prompt commencement of antimalarial therapy, splenectomy has long been recommended for patients with malaria-induced spleen rupture. Many now advocate for conservative management, including in patients with unstable hemodynamics, with strict bed rest, fluid management, transfusions if needed, and close clinical and hematological observation for at least two weeks in a hospital with surgical and intensive care availability [1]. When surgery is unavoidable, conservative approaches (splenic repair or partial excision) are often preferred, leaving total splenectomy as a last resort.

Our study has some limitations: we present here a single case with only one extra-splenic large blood vessel available for comparison. Our observations need to be confirmed in similar cases. Nevertheless, this case report reinforces the importance of furthering studies of intrasplenic infections to determine to what extent they contribute to the burden of human malarias with a tropism for reticulocytes, and better understand ovale malaria pathogenesis and potential clinical complications.

## Supporting information

S1 Table Demographic and clinical characteristics of patients with spontaneous splenic rupture related to *Plasmodium ovale sensu lato* malaria. *DRC: Democratic Republic of Congo.
(DOCX)

S1 File Raw data. (XLSX)
