# Supplementary material for: Splenic accumulation of intact Plasmodium ovale sensu lato-infected red blood cells in a patient presenting with splenic rupture
Source: PLoS Negl Trop Dis. 2026 Jan 8;20(1):e0013897. doi: 10.1371/journal.pntd.0013897 (PMC12829925; doi:10.1371/journal.pntd.0013897)
Supplement: S1 Table — *DRC: Democratic Republic of Congo. (DOCX) [file pntd.0013897.s001.docx]

|  | [2] **Facer et Rouse (1991)** | [3] **Lemmerer et al. (2016)** | [4] **Mouline et al. (2017)** | [5] **Zidouh et al. (2017)** | [6] **Wankap et al. (2020)** | **Benabdelmoumen et al. (this case)** |
| --- | --- | --- | --- | --- | --- | --- |
| **Age (years), sex** | 51, Female | 29, Male | 34, Male | 42, Male | 41, Female | 35, Male |
| **Medical history, treatments** | Unknown | None | None | None | None | None |
| **Country visited, length of stay** | Ghana, 19 days | DRC*, 1 month | Ivory Coast, 6 months | Central African Republic, N/A | Mali, Guinea Conakry, unknown | Chad, 4 months  Senegal, N/A |
| **Reason of travel** | Tourism | Business | Unknown | Expatriate | Migrant | Humanitarian aid-worker, Expatriate |
| **Malaria chemoprophylaxis** | None | None | Mefloquine | Mefloquine | None | Atovaquone/Proguanil (in Chad only) |
| **Initial clinical presentation:**   - **Fever** - **Headaches** - **Abdominal pain** - **Vomiting** - **Diarrhea** - **Splenomegaly** | +  +  -  -  -  Unknown | +  +  +  -  -  Unknown | +  -  -  +  -  - | +  -  +  +  -  + | +  -  +  +  +  + | +  +  +  +  -  + |
| **Initial laboratory work:**   - **Hb (g/dL)** - **Platelets (x10^9^ /L)** - **Parasitemia (%)** | Unknown  Unknown  1.8 | 10.3  65  Unknown | 11.2  134  2 | 11  77  0.01 | 9.2  93  3 | Unknown  56  0.9 |
| **Time between onset of symptoms and splenic rupture** | 2 days | 9 days | Unknown | Unknown | 30 days | 10 days |
| **Time between start of treatment and splenic rupture** | No treatment, post-mortem diagnosis | 48 hours | 48 hours | Unknown | Concomitant | 2 hours |
| **Treatment (splenectomy or conservative treatment)** | No treatment, post-mortem diagnosis | Splenectomy | Splenectomy | Splenectomy | Splenectomy | Splenectomy |
| **Outcome** | Death | Favorable | Favorable | Favorable | Favorable | Favorable |

**Supplementary data**

**Supp table 1.** **Demographic and clinical characteristics of patients with spontaneous splenic rupture related to *Plasmodium ovale* malaria**

*DRC: Democratic Republic of Congo
